# COVID-19 in Ethiopia: A contextual approach to explaining its slow growth

**DOI:** 10.7189/jogh.10.020369

**Published:** 2020-12

**Authors:** Dawit Wondimagegn, Adane Petros, Yidnekachew Asrat, Tesfamariam Aklilu, Abiy Seifu Estifanos, Adamu Addissie, Wondwossen Amogne, Tewodros Haile Gebremariam, Carrie Cartmill, Cynthia Whitehead, Sophie Soklaridis, Helen Yifter

**Affiliations:** 1 Department of Psychiatry, School of Medicine, College of Health Sciences, Addis Ababa University, Addis Ababa, Ethiopia; 2 Department of Internal Medicine, School of Medicine, College of Health Sciences, Addis Ababa University, Addis Ababa, Ethiopia; 3 Department of Reproductive, Family and Population Health, School of Health Sciences, Addis Ababa University, Addis Ababa, Ethiopia; 4 Department of Preventive Medicine, School of Public Health, College of Health Sciences, Addis Ababa University, Addis Ababa, Ethiopia; 5 The Wilson Centre, University Health Network, University of Toronto, Toronto, Ontario, Canada; 6 Department of Family & Community Medicine, University of Toronto, Toronto, Ontario, Canada.; 7 Women’s College Hospital, Toronto, Ontario, Canada; 8 Department of Psychiatry, University of Toronto, Toronto, Ontario, Canada; 9 Centre for Addiction and Mental Health, Toronto, Ontario, Canada

The COVID-19 pandemic is a time of many predictions and rapid learning. Early on, the World Health Organization (WHO) identified Ethiopia as one of thirteen countries in Africa as a top priority for COVID-19 preparedness [1]. Surprisingly and thankfully, while an early catastrophic outbreak was feared, the rise in COVID-19 cases in Ethiopia has continued to be slow, with few significant health systems impacts or changes in the patterns of morbidity and mortality [2]. Despite its high-risk status, over four months after is first confirmed case, Ethiopia, with a population of 110 million, reported only 8181 cases of COVID-19 on July 15th, 2020, or 74.37 cases per 1 million people [2]. In comparison, the United States, with a population three times that of Ethiopia, had over 2 million cases within four months of its first case [3]. Brazil, with a population twice the size of Ethiopia, had over 1 million cases within four months of its first case, and South Africa, with a population half that of Ethiopia, had over 150 000 cases within four months of its first case [3].

The complex interplay between biology, health policy, health human resources, sociocultural factors, and political decision-making influence pandemic outcomes, although these interactions are challenging to track. Nations with seemingly similar sociocultural and political structures have taken drastically different measures to address COVID-19, suggesting that even within large geographical regions, local context matters [4]. Sub-Saharan Africa is showing similar variability between nations, and there is acknowledgement that a one-size-fits-all approach is inadequate for a continent with significant cultural, political, economic, and resource diversity [5]. As of now—in spite of an outpouring of academic writing on comparative COVID-19 experiences—there is a relative dearth of literature led by Africans [6]. It is imperative that the perspectives and expertise of African academics are included in global conversations, as they may be highly relevant for other nations with significant population and contextual diversity. In this commentary, we describe how we developed a context-specific approach to gathering and analyzing data to help explain the intersection of numerous variables in influencing the ongoing slow growth of COVID-19 in Ethiopia.

## CONTEXTUAL APPROACH

We employed a three-step process early in the pandemic to understand the pattern of COVID-19 in Ethiopia: a focus group to explore explanatory themes, an analysis of current evidence, and a panel discussion with a larger audience of experts. The single focus group was conducted on April 25, 2020 and consisted of in-country experts including those with clinical, public health and health system leadership and policy backgrounds, who are actively engaged in the COVID-19 pandemic control program at institutional and national levels. The goal of the focus group discussion was to draw upon the multi-faceted perspectives of participants to identify explanations and rationales for Ethiopia’s low COVID-19 rate. Second, an extensive search of the academic literature was done to identify evidence for the identified explanatory themes. We searched Medline, Embase and the Chinese Biomedical Literature Database using keywords identified in the focus group without limiting our selection to specific study designs. We also searched for pre-print publications from medRxiv. Finally, we combined information from the focus group and the literature review to engage with a multidisciplinary panel of over 80 high level academic and policy experts and health professionals from Ethiopia on May 8, 2020. The aim of this two-hour virtual panel discussion was to validate findings from the focus group and literature review and to develop recommendations for the ongoing monitoring and tracking of COVID-19 in Ethiopia. During the panel discussion, first, the results of the focus group and literature synthesis were presented; second, a senior infectious disease expert, pulmonologist and epidemiologist provided feedback on findings; and finally, feedback and input from the remaining discussion participants of the panel was taken.

The focus group discussion identified 30 possible contributors to the status of the epidemic in Ethiopia. These possible contributors were then categorized into four potential explanatory interpretations (see **Table 1**). Each explanatory interpretation was tested against current evidence identified in the literature, as summarized below.

**Table 1:** Possible contributors to low counts of COVID-19 cases in Ethiopia, based on a focus group discussion on April 25, 2020

Potential contributing factors	Explanatory interpretation
Reliance on self and institutional reporting	Testing-related factors
Insufficient disclosure	
Insufficient contract tracing	
Prolonged incubation period and asymptomatic viral shedding	
Low testing capacity	
Narrow case definition	
Challenges with specimen collection and transportation	
Insufficient training and experience	
Standardization of testing for local use	
Low adjuvant investigations using tests other than RT-PCR	
Low sensitivity of RT-PCR in early stages of infection	
High prevalence of other infectious diseases provides protection	Differences in host-agent factors
Cross-immunity with other viruses	
Genetic diversity of both virus and host	
Environmental factors (temperature, humidity, altitude)	
Age distribution	
Urban to rural ratio	
Social determinants (culture, diet, pets)	
Hygiene hypothesis (including immunizations)	
Travel and movement patterns	
General naiveté for modern medicine	
Timing and early implementation of interventions	Early implementation of public health measures
Quarantine	
Isolation	
Banning of mass gatherings	
Border closures	
School closures	
Health education campaigns	
Ethiopia is in its 8^th^ week of monitoring	The epidemic trajectory
Ethiopia is in the pre-growth phase of infections	

**Figure:**
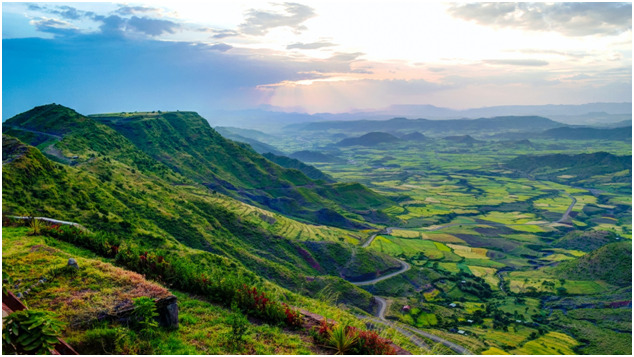
Photo: The landscape and context of Ethiopia. From: https://www.istockphoto.com/photo/panorama-of-semien-mountains-and-valley-around-lalibela-ethiopia-gm672077498-123061719.

### Testing-related factors

The first possible explanation proposed that Ethiopia’s low case count was a result of insufficient testing to identify cases. Country testing status can be evaluated in terms of three different variables: testing coverage (number of tests per thousand population), number of tests performed for each confirmed case, and case fatality rate. All three variables can help us understand the true spread of the virus [7]. The number of tests per confirmed case is arguably the most helpful, given that a smaller outbreak requires less testing. Like other countries, Ethiopia’s test coverage has improved over the course of the pandemic. Early on it had low coverage with approximately 0.14 tests per thousand population being conducted during May, 2020. Still, it has consistently had one of the better figures for the number of tests performed per confirmed case, with 31 tests for each confirmed case currently being conducted [3]. This is much higher than countries with a high burden of COVID-19. For example, 12.4 tests per confirmed case are currently being performed in the USA, 2.3 tests per confirmed case in Brazil, and 6.6 tests per confirmed case in South Africa [3]. Ethiopia’s ratio of tests performed for each confirmed case has consistently argued for Ethiopia’s overall low prevalence of COVID-19 cases thus far.

### Host-agent factors

The second possible explanation proposed that the Ethiopian population may have more immunity and/or lower susceptibility to COVID-19. Preliminary evidence suggested that Ethiopia may have lower susceptibility for the exponential transmission of COVID-19. Potential contributors to the slow spread of COVID-19 in the country (and across the sub-Saharan region) were that Ethiopia is a tropical country with high average temperatures; the capital city is at high altitude; the high prevalence of tuberculosis and malaria may have a negative association with COVID-19 [8]; a universal Bacillus Calmette-Guérin vaccination program [8]; a high rural to urban population ratio; a young median age; low travel and movement within the country and across the globe; and patient derived genetic mutations of the virus may be protective [9].

### Early public health interventions

The third possible explanation proposed that the early implementation of non-pharmacological public health interventions (NPIs) in Ethiopia has slowed the progression of the epidemic. These early interventions included mandatory quarantining of passengers from abroad, closing borders to prevent travel in and out of the country, closing schools, banning mass gatherings, and early health education campaigns [10]. Each of these may have helped slow COVID-19 transmission. It is still too early to identify the specific public health measures that have been influential. Measuring the impact of public health interventions takes time, requires measurement of compliance, and can be difficult to track at a population level. Because of this difficulty, it is not possible to rule out the role of NPIs in explaining the slow growth of COVID-19 in Ethiopia.

### The epidemic trajectory

The fourth possible explanation proposed that Ethiopia remains in a different phase of the epidemiological curve than elsewhere. Little is known about the initial phases of the epidemiological curve in countries that experienced earlier outbreaks, and to what extent cases were present but undetected. It is also possible that Ethiopia has experienced a different shape in the epidemiological curve with a longer and flatter first wave. However, observation in other countries has suggested that while the rate of growth may be variable, increased cases is the norm. While the rate of growth has been recently increasing, and COVID-19 cases may well eventually catch up with the rest of the world, it is noteworthy that Ethiopia has shown a sustained pattern of slow growth over an extended period of time.

## PANEL DISCUSSION AND CONCLUDING REMARKS

During the panel discussion the three invited panelists (senior infectious disease expert, pulmonologist and epidemiologist) and the panel discussion participants corroborated and validated the literature synthesis findings. The overall consensus was that Ethiopia has experienced a slow and delayed epidemic trajectory because of the combined contributions and interaction of testing-related factors, contextual host-agent dynamic and NPIs. Despite this slow growth, the panel accordingly recommended that it would be extremely unwise to risk complacency, and ongoing monitoring of all epidemiological indicators is essential. Policy makers should continue to advance a coordinated strategy to reduce the impact of COVID-19 should it become more severe at later stages. This approach, which brought together infectious disease, pulmonology and epidemiology experts from public health and academic institutions, provided insight and direction for making reasoned decisions that incorporated contextual realities within Ethiopia. We hope that other countries with population and contextual diversity might learn from our strategies in ways that allow them to prepare for the unpredictable future phases of COVID-19.
